# Editorial: Concussion and Sport: A Sociocultural Perspective

**DOI:** 10.3389/fspor.2021.754002

**Published:** 2021-09-13

**Authors:** Dominic Malcolm, Alison Doherty, Jimmy Sanderson, Kathleen Bachynski

**Affiliations:** ^1^School of Sport, Exercise and Health Sciences, Loughborough University, Loughborough, United Kingdom; ^2^School of Kinesiology, Western University, London, ON, Canada; ^3^Department of Kinesiology and Sport Management, Texas Tech University, Lubbock, TX, United States; ^4^Muhlenberg College, Allentown, PA, United States

**Keywords:** concussion, public health, brain injury, sociology discipline, management

The Research Topic *Concussion and sport: a sociocultural perspective* is inextricably tied to the COVID-19 pandemic. Partly this relates to timing, as COVID-19 became the topic of conversation through which we (the editorial team) came to forge our working relationship. Editorial meetings invariably began with comparisons of transmission rates, restrictions on social contact and latterly vaccine up-take in our respective parts of the world. But there are deeper parallels, between COVID-19 and brain injury in sport as public health issues. In this article we draw attention to these parallels as a way of introducing some key sociocultural aspects of concussion in sport as a public health crisis.

For a number of years, authors of both populist texts (Carroll and Rosner, 2011) and medical journal articles (Zemek et al., 2016) have described concussion as an epidemic. More recently, concerns about concussion have become global in scope, inviting reclassification to pandemic (Malcolm, 2020). While it is important to draw a distinction between an epidemic/pandemic as technically defined—notably concussion is neither a disease nor infectious—increasingly, non-communicable diseases (NCD) such as heart disease and cancer have been shown to have characteristics of transmissibility; they can be passed along “*via* social networks, the built environment, social and economic conditions, and intergenerational transmission” (Allen, 2017, p. 6). Thus, “There is much to gain from viewing the rise of preventable NCD mortality and morbidity as a pandemic” (Allen, 2017, p. 8). The temporally specific structural features of the society in which any public health crisis arises crucially influence the way these conditions are experienced.

The importance of understanding the historical, social, economic and political factors specific to a pandemic are equally evident in our understanding of the transmission of COVID-19 and the phenomenon of brain injury in sport. For instance, the impact of COVID-19 is fundamentally linked to the degree of global interconnectedness (of particular nations) at this particular point in human history, and the impact of concussion across sport is similarly shaped by contemporary modes of communication and sports fandom. It has become something of a truism in COVID times to say that the pandemic has accelerated social trends that were already evident (e.g., the move toward online retail) and exposed the fault lines or weaknesses in our societies that already existed (e.g., the greater impact on already disadvantaged populations). Similarly, it could be said that debates about concussion in sport are the latest in a long series of concerns about the violence of certain sports, but debates that have exposed as never before the risk culture of sports and the asymmetric power relations between players, coaches and medical staff.

However, curating *Concussion and Sport: a sociocultural perspective* at a time when public health responses to pandemics have been brought into such sharp relief has also enabled us to see more acutely the ethical, scientific and political dynamics of debates about concussion.

- At an ethical level, the state's response to COVID-19 posed several conundrums that equally resonate with public health debates around concussion: to what extent can society-wide public health concerns justify constraints on individual liberties? What processes should be involved, and which people or organizations should be responsible for striking the balance between protecting life and respecting individual autonomy when these ethical values come into tension? Do the answers to these questions vary according to social, economic and political context? To what extent should public policy prioritize the protection of those at greatest risk for both short-term and long-term harms? COVID-19 and concussion both present the challenge of navigating how various forms of physical contact can become inherently dangerous to health while simultaneously also being the source of profound social meaning for individuals.
- Research-based responses to the COVID-19 pandemic have revealed some valuable lessons for the scientific management of concussion. First, the imperfections of epidemiological data became writ large. Debates about the relative merits of assessing deaths with COVID or deaths from COVID, or excess deaths compared to periodic averages, have parallels in debates around the quantification of the incidence of concussion in sport, and between different demographic cohorts. The oft-cited CDC estimate of 1.6–3.8 million sport-related concussions per year is indicative of the underlying limitations in “objectively” quantifying concussion. Second, the complexities of introducing technological solutions to health problems have become increasingly apparent. In places where vaccines have been widely administered we see that this represents the beginning of a new phase rather than the solution to the pandemic. Similarly, the development of more accurate measures of physical impact, or reliable diagnostic biomarkers, will only change rather than resolve the issues around concussion (Bachynski and Goldberg, 2014). Moreover, they will entail complex questions of implementation, distribution and social justice.
- COVID-19 policy interventions similarly revealed some of the complexities of managing modern societies which responses to concussion must confront. In both cases it is apparent that education does not directly lead to behavioral change, and there is a sense of frustration at the failure of the public to comply with measures implemented for their own health. In both cases the difficulties of managing the distinct needs of different generations come to the forefront, as concerns about the health of older generations (their greater vulnerability to COVID-19, the manifestation of dementia in the retired sporting population) invoke restrictions to the lifestyles of the young who are often perceived as less affected by these health concerns. At the same time, in both cases children also have their own unique vulnerabilities, from MIS-C (multisystem inflammatory syndrome in children) among pediatric COVID-19 cases to the harms of repeated trauma to still-developing brains. Both cases manifest the importance and complexity of using epidemiological methods to trace the connections between acute exposure and long-term health effects. Physical and mental health issues are intricately linked, with the detrimental mental health impact of restrictions on social gatherings and post-concussive syndrome juxtaposed against the apparent necessity of physical activity and sport for positive mental health. Finally, the cases of COVID-19 and concussion each highlight the challenges that the increasing political polarization of contemporary societies brings to human attempts to manage social affairs, including the prominence of “fake news” and accusations of political manipulation of scientific “fact” (Malcolm, 2021).

The contributions to this Research Topic speak to and illuminate the social and political dimensions of the public health response to concussion in sport in various ways. In article 1, “The Tragedy of the Punch Drunk”: Reading Concussion in Australian Sporting Newspapers, 1803–1954, Townsend provides a historical analysis of trends in discourse surrounding concussion in the Australian sporting press. His study suggests both striking similarities over time as well as distinguishing features of contemporary debates around concussion, raising questions about the political and institutional factors that either prevent or facilitate the emergence of broader public concern over brain trauma in sport. In article 2, “Learning to listen to them and ask the right questions.” Bennet Omalu, scientific objectivities, and the witnessing of a concussion crisis, Hollin explores issues around the scientific knowledge underpinning concussion. Here is illustrated how perhaps *the* crucial or foundational scientific discovery in the concussion crisis relies upon somewhat unconventional notions of objectivity, and in particular a fusing of religiosity and experimental methodologies.

Articles 3 and 4 highlight issues around education and public knowledge. In Athletes' and Coaches' Attitudes Toward Protective Headgear as Concussion and Head Injury Prevention: A Scoping Review, Tjønndal and Wågan conclude that: (a) athletes have (misplaced) confidence in the degree of protection offered; and (b) that regardless of these beliefs, they tend to be resistant to the use of protective headgear in sport. Next, Lindner and Hawkins, in their article Education, Political Party, and Football Viewership Predict Americans' Attention to News About Concussions in Sports, illustrate how an understanding of concussion is not only mediated by prior athletic experience, but also political orientation. They find that more left-leaning (in this case Democrat-aligned) members of the public express greater concerns about concussion, and greater trust in the emerging scientific evidence about the risks of participating in certain sports.

Our final two articles focus on regulatory issues. In article 5, Two Sports, Two Systems, One Goal: A Comparative Study of Concussion Policies and Practices of the Australian Football League and Hockey Canada, Greenhow and Doherty identify common themes and divergent practices in current regulatory approaches to concussion across two leading sport institutions in different countries. They conclude that historical and cultural roots frame the regulatory legitimacy of these institutional actors as they direct the agenda to manage and mitigate harm associated with concussion in their respective sports and nations. Finally, in their article Concussion Reporting and Safeguarding Policy Development in British American Football: An Essential Agenda, Travis et al. take a more micro-oriented perspective, drawing on the responses of players and club administrators to demonstrate shortcomings in the welfare policies of British American Football. They note that medical support for players is often poor which, in turn, has ramifications for the diagnosis and management of players' return to play from injury and thus conclude that the sport's concussion guidelines are insufficient to ensure the organizational goal of providing a safe and enjoyable playing environment.

Our goal for this collection was to highlight the fundamental role of social science in informing the “concussion crisis” in sport (Malcolm, 2020). Through this we wanted to provide a forum for research that would advance and expand the knowledge base which informs existing public health interventions. We believe that individually and collectively the articles in this special topic significantly advance this effort, and provide a springboard for the continued consideration of sociocultural perspectives on concussion. In parallel, the COVID-19 pandemic has shown, perhaps as never before, how the major issues confronting humanity require multi- and interdisciplinary insights and solutions. In the forthcoming years it is hoped that lessons about the importance of collaborative cross-paradigmatic science become part of the post-COVID-19 “new normal,” and inform the way scholars and practitioners seek to negotiate the challenges posed by concussion in sport.

## Author Contributions

DM wrote the first draft of this Editorial. All authors viewed and returned comments on the first draft, viewed, and confirmed agreement with the second and final draft.

## Conflict of Interest

The authors declare that the research was conducted in the absence of any commercial or financial relationships that could be construed as a potential conflict of interest.

## Publisher's Note

All claims expressed in this article are solely those of the authors and do not necessarily represent those of their affiliated organizations, or those of the publisher, the editors and the reviewers. Any product that may be evaluated in this article, or claim that may be made by its manufacturer, is not guaranteed or endorsed by the publisher.
